# Achieving a physiological cortisol profile with once-daily dual-release hydrocortisone: a pharmacokinetic study

**DOI:** 10.1530/EJE-15-1212

**Published:** 2016-07-01

**Authors:** Gudmundur Johannsson, Hans Lennernäs, Claudio Marelli, Kevin Rockich, Stanko Skrtic

**Affiliations:** 1 Department of EndocrinologyInstitute of Medicine, Sahlgrenska Academy, University of Gothenburg, Gothenburg, Sweden; 2 Department of PharmacyUppsala University, Uppsala, Sweden; 3 Shire International GmbHZug, Switzerland; 4 Shire PLCWayne, Pennsylvania, USA; 5 AstraZeneca R&DMölndal, Sweden

## Abstract

**Objective:**

Oral once-daily dual-release hydrocortisone (DR-HC) replacement therapy was developed to provide a cortisol exposure−time profile that closely resembles the physiological cortisol profile. This study aimed to characterize single-dose pharmacokinetics (PK) of DR-HC 5–20mg and assess intrasubject variability.

**Methods:**

Thirty-one healthy Japanese or non-Hispanic Caucasian volunteers aged 20−55 years participated in this randomized, open-label, PK study. Single doses of DR-HC 5, 15 (3×5), and 20mg were administered orally after an overnight fast and suppression of endogenous cortisol secretion. After estimating the endogenous cortisol profile, PK of DR-HC over 24h were evaluated to assess dose proportionality and impact of ethnicity. Plasma cortisol concentrations were analyzed using liquid chromatography−tandem mass spectrometry. PK parameters were calculated from individual cortisol concentration−time profiles.

**Results:**

DR-HC 20mg provided higher than endogenous cortisol plasma concentrations 0−4h post-dose but similar concentrations later in the profile. Cortisol concentrations and PK exposure parameters increased with increasing doses. Mean maximal serum concentration (C_max_) was 82.0 and 178.1ng/mL, while mean area under the concentration−time curve (AUC)_0−∞_ was 562.8 and 1180.8h×ng/mL with DR-HC 5 and 20mg respectively. Within-subject PK variability was low (<15%) for DR-HC 20mg. All exposure PK parameters were less than dose proportional (slope <1). PK differences between ethnicities were explained by body weight differences.

**Conclusions:**

DR-HC replacement resembles the daily normal cortisol profile. Within-subject day-to-day PK variability was low, underpinning the safety of DR-HC for replacement therapy. DR-HC PK were less than dose proportional – an important consideration when managing intercurrent illness in patients with adrenal insufficiency.

## Introduction

Adrenal insufficiency (AI) is a life-threatening orphan disease that requires daily glucocorticoid replacement to increase cortisol concentrations to pre-disease values (1, 2). Hydrocortisone is the most commonly used glucocorticoid replacement therapy and requires multiple daily oral administrations to provide an adequate cortisol concentration−time profile (1). In a typical dosing regimen, hydrocortisone is taken two or three times per day, with the highest dose in the morning, a lower dose at midday and, if necessary, a third dose in the late afternoon or early evening (3). This conventional treatment regimen has demonstrated efficacy in AI but long-term clinical outcomes remain unsatisfactory, with higher premature mortality and morbidity and impaired quality of life (QoL) compared with the general population (4, 5, 6, 7, 8). Large studies have shown that the all-cause mortality risk is more than two-fold higher in patients with primary AI than in the general population, mainly resulting from cardiovascular, malignant, and infectious diseases (4, 5). Furthermore, patients with chronic AI often experience adrenal crisis despite treatment and education, with an associated mortality rate of 6.3% (9).

The inability of conventional glucocorticoid replacement to provide a plasma cortisol exposure−time profile that resembles the circadian physiological cortisol profile is likely to have direct adverse effects on metabolism, general health, and QoL (1, 3). Higher than physiological cortisol exposure in the late afternoon and evening may be a particularly important cause of metabolic dysfunction and altered sleep patterns, but excessive total glucocorticoid exposure and inadequate rescue therapy during intercurrent illness will also have important clinical implications, including increased morbidity and mortality related to infectious disease and increased cardiometabolic risk (1, 3, 6, 9, 10, 11, 12, 13, 14). In a study in hypopituitary patients, glucocorticoid replacement therapy was associated with dose-related increases in BMI, triglycerides, low-density lipoprotein cholesterol, and total cholesterol (12). Abnormally high cortisol levels in the evening have been associated with insomnia and increased sleep disturbance (11, 14).

Modern biopharmaceutical techniques have allowed the development of a once-daily, dual-release hydrocortisone (DR-HC) tablet, comprising an immediate-release coating and an extended-release core, for oral glucocorticoid replacement therapy in AI (15). This dual-release formulation was developed to provide a cortisol exposure−time profile that more closely resembles the physiological serum cortisol profile than provided by standard formulations (16). Clinical trials in healthy volunteers and patients with AI have demonstrated that DR-HC produces a diurnal plasma cortisol profile that mimics the daytime circadian-based physiological serum cortisol profile more closely than conventional glucocorticoid replacement therapy with immediate-release tablets (15, 16). The study conducted in patients with AI also showed that treatment with once-daily DR-HC tablets significantly improved metabolic factors and QoL compared with conventional hydrocortisone tablets administered three times per day (16). Furthermore, a prospective trial in patients with AI showed that DR-HC significantly reduced BMI and hemoglobin A1c (HbA1c) and stabilized QoL compared with conventional hydrocortisone therapy (17).

This study was conducted to expand the pharmacokinetic (PK) data of DR-HC to provide support for dosing recommendations. Hence, the main study objectives were to characterize the single-dose plasma PK of DR-HC tablets across the dose range of 5−20mg in the fasted state in healthy volunteers and to assess intrasubject variability.

## Subjects and methods

### Subjects

Healthy men and women aged 20−55 years with a BMI 18−30kg/m^2^, body weight of ≥50kg for men and ≥45kg for women, and who were of either Japanese descent or non-Hispanic Caucasian were included in the study. Exclusion criteria included: any known metabolic or endocrine disorders; clinical or laboratory signs of significant cerebral, cardiovascular, respiratory, renal, hepatobiliary, pancreatic, or gastrointestinal emptying/motility disease that could interfere with study assessments or completion; history of hydrocortisone intolerance; hypersensitivity to DR-HC, dexamethasone, or any of their excipients; systemic fungal infection; hepatitis B or C infection; known immune deficiency virus; taken any investigational study drug within 30 days of starting the study; drug or alcohol abuse within 1 year of starting the study. Any agent that may interfere with hydrocortisone PK or hepatic drug metabolizing capacity within 14 days before starting the study were not permitted. Pregnant or lactating women were not eligible for the trial.

All volunteers provided written informed consent before entering the study. The local Ethics Committee approved the study protocol and the study was performed according to the principles of Good Clinical Practice and the Declaration of Helsinki. The trial was conducted between 15 November 2013 and 21 December 2013.

### Study design

This was a randomized, open-label, four-period crossover, single-dose PK study of oral DR-HC tablets conducted at a single center. All subjects were screened within 21 days before the first treatment period and had a baseline 24-h assessment of endogenous cortisol secretion, performed after overnight fast and before the first treatment period. Eligible subjects were then randomized to one of four treatment sequences, each comprising four 3-day treatment periods separated by wash-out periods of at least 72h. Blood samples for PK assessments were collected at 15, 30, 45, 60, 90, 120, and 150min, hourly from 3 to 10h, and at 12, 15, and 24h after dosing with study drug in order to provide evaluable concentration−time profiles. Subjects were provided with standardized meals throughout the study.

Medical history, physical examination, vital signs, and medication history were assessed at baseline. Subjects were monitored throughout the study for adverse events (AEs). Subjects returned to the clinical trial unit approximately 1 week after the final dose for a follow-up assessment, which included a physical examination, vital signs, ECG, and routine laboratory parameters.

### Interventions

Oral dexamethasone 1mg was administered at 18:00 and 23:00h (±15min) on day 1 and at 07:00, 11:00, 17:00, and 23:00h (±15min) on day 2 of each treatment period to suppress endogenous cortisol secretion during PK sampling (18). Endogenous cortisol suppression was defined as a pre-dose plasma cortisol concentration ≤50nmol/L (within 15min before 08:00h on day 2).

Single doses of DR-HC produced at different manufacturing sites (Recipharm, Stockholm, Sweden (test site) and Galenica, Malmö, Sweden (reference site)) were evaluated for bioequivalence. Doses of 5, 15 (3×5), and 20mg (test site) and 20mg (reference site) were administered orally once daily at 08:00h on day 2 of the treatment period after overnight fast. Assuming that bioequivalence of DR-HC manufactured at the different sites is established, administration of the two 20mg doses of DR-HC enabled the evaluation of intrasubject variability in PK.

### Analytical methods

Plasma cortisol concentrations were analyzed using a validated liquid chromatography−tandem mass spectrometry (LC-MS/MS) method with an assay range of 1−500ng/mL (Quotient Bio Analytical Sciences, Cambridgeshire, UK). The lower limit of quantification (LLOQ) was 1ng/mL and the precision of the assay (coefficient of variation) was ≤5.7%.

### Statistical analysis

All PK analyses were performed on the PK population, which included all subjects with at least one evaluable concentration−time profile. There were two definitions for an evaluable concentration−time profile: protocol defined and PK analysis plan (PKAP) defined, the latter being more conservative. Both definitions of an evaluable concentration−time profile included the following criteria: >50% of samples with values above the LLOQ, a pre-dose plasma cortisol concentration ≤50nmol/L (indicating dexamethasone suppression), and no major deviations related to investigational product intake (e.g. vomiting) or PK sampling. The PKAP-defined evaluable PK profile also had to have a good estimate of the terminal elimination phase, that is, an adjusted *R*^2^ goodness-of-fit statistic >80%, and sufficient data to estimate the area under the concentration−time curve (AUC) with ≤10% of the integrand extrapolated. Data for the protocol-defined evaluable concentration−time profiles are primarily presented here. Demographics and baseline characteristics were summarized for the intent-to-treat (ITT) population, which included all subjects who received the study drug.

PK analyses for hydrocortisone (cortisol) were determined by non-compartmental analysis using WinNonlin version 6.2 or higher (Pharsight Corp., St Louis, MO, USA). PK parameters were calculated from individual cortisol concentration−time profiles and individual baseline-corrected cortisol concentration−time profiles. The baseline-corrected concentration was calculated by subtracting the baseline value (measured within 15min before treatment) from each concentration in each individual plasma concentration−time profile.

All plasma AUC parameters were calculated using linear/logarithmic trapezoidal method. AUC_0−∞_ was calculated by extrapolating the curve to infinity using the sum of AUC_last_+C_last_/λ_z_, where AUC_last_ is the AUC through to the last measurable concentration, C_last_ is the last measurable concentration, and λ_z_ is the terminal rate determined using goodness-of-fit statistics and an adequate number of points in the terminal phase of the curve. AUC_0−12h_ was calculated from time zero to the sample taken at 12h after dosing. The terminal half-life was obtained from the elimination rate constant as ln2/λ_z_. Summary statistics were determined for all PK parameters by treatment and for the endogenous cortisol plasma assessment at each time point.

Bioequivalence, defined as the 90% confidence interval (CI) being within the 80−125% limits, was determined for the two 20mg tablets (from the test and reference sites) using the average bioequivalence approach (two one-sided tests procedure) (19, 20). Within-subject variability was examined with log-transformed maximal cortisol plasma concentration (C_max_), AUC_0−12h_, and AUC_last_ (uncorrected for pre-dose baseline cortisol concentrations). Dose proportionality of baseline-corrected and uncorrected PK parameters over the administered dose range (test site) was examined using the power model method, with ethnicity as a potential covariate.

## Results

### Baseline characteristics

Thirty-one patients were randomized and included in the ITT and PK analysis populations. Thirty subjects (97%) completed the study; one subject withdrew for personal reasons after receiving two doses. The mean age of the study subjects was 38.5 years (range, 22−54 years), mean body weight was 69.3kg (range, 48.8−84.7kg), mean BMI was 23.7kg/m^2^ for men and 21.9kg/m^2^ for women, 51.6% were of Japanese descent and 48.4% were Caucasian (Table 1). The Japanese and Caucasian groups were well matched for age, BMI, and sex, but the Japanese group had a lower mean (s.d.) weight of 66.5 (9.7)kg compared with the Caucasian group of 72.2 (9.6)kg due to two outliers in the Japanese group with very low weights (48.8 and 52.4kg).

**Table 1:** Demographic and baseline characteristics: ITT population. Results are presented as *n* (%) for categorical variables and mean (s.d.) for continuous variables.

	**ITT** (*n*=31)
Age (years)	38.5 (10.1)
Sex	
Male	26 (83.9%)
Female	5 (16.1%)
Body weight (kg)	69.3 (9.9)
BMI (kg/m^2^)	
Male	23.7 (1.7)
Female	21.9 (2.6)
Race	
Non-Hispanic Caucasian	15 (48.4%)
Japanese	16 (51.6%)
Clinically relevant disease	10 (32.3%)

ITT, intent-to-treat.

More subjects had protocol- versus PKAP-defined evaluable concentration−time profiles. For uncorrected PK parameters with the two 20mg tablets, 23 (77%) and 19 (63%) subjects had evaluable PK profiles using the PKAP definition, while 29 (97%) and 30 (100%) subjects were evaluable using the protocol definition. The difference between the two populations was the inclusion of subjects that had a poorly estimated λ_z_, as indicated by an adjusted *R*^2^ of ≤0.8, due to the presence of endogenous cortisol concentrations and the inability to fully correct for changing levels throughout the profile.

### Comparison of replacement hydrocortisone with endogenous cortisol profile

**Figure 1:**
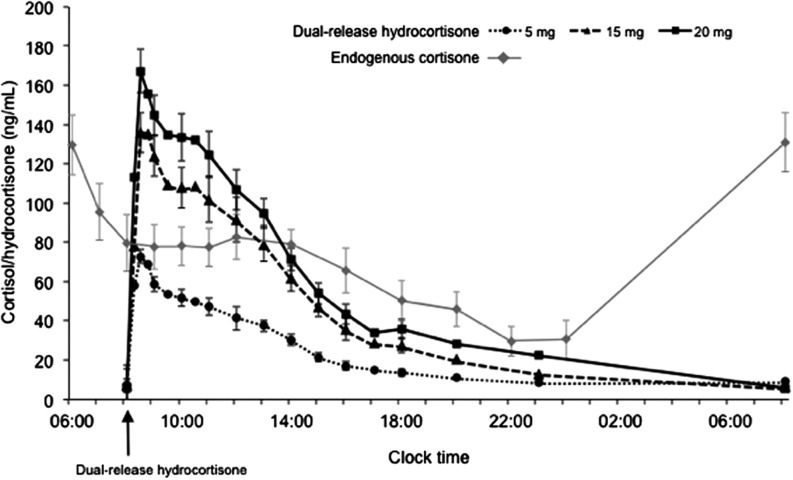
Mean (95% CI) plasma concentration−time profiles for endogenous cortisol and after single oral doses of DR-HC 5, 15, and 20mg (test site) in healthy subjects in the PK population. Endogenous cortisol concentrations were not assessed after 00:00 until 06:00 on day 2.

Oral replacement treatment with DR-HC 20mg seemed to provide higher than endogenous cortisol plasma concentrations 0−4h post-dose, with the peak concentration measured at 0.5h after dosing, but similar plasma concentrations later in the profile, particularly from midday to the last evaluations in the day at 23:00h (after DR-HC administration) and midnight (endogenous cortisol) (Fig. 1). There was considerable intersubject variability in both the endogenous cortisol plasma profile and the cortisol plasma concentrations following dosing with DR-HC. For the first measurement of endogenous cortisol concentration at 06:00h on day 1, values ranged between 20.4 and 190.0ng/mL, while midnight levels ranged between 9.6 and 105.0ng/mL and levels at 08:00 h on day 2 ranged from 53.1 to 194.0ng/mL; all subjects achieved peak plasma concentrations of ≥95ng/mL during the 24-h evaluation of endogenous cortisol. Peak cortisol plasma concentrations 30min after administration of DR-HC 20mg (test site) ranged from 120.0 to 266.0ng/mL, while at 23:00h concentrations had diminished to 0−65.0ng/mL. AUC_last_ following dosing with DR-HC 20mg (test site) ranged from 836.3 to 1670.9h×ng/mL.

### Plasma PK parameters for DR-HC

Cortisol plasma concentrations and hydrocortisone PK exposure parameters increased with increasing doses of DR-HC, and a biphasic release profile was observed (Fig. 1 and Table 2). Plasma cortisol/hydrocortisone concentrations remained above baseline levels for at least 15h after all doses. The percentage of exposure extrapolated in the plasma concentration−time profile (% extrapolated AUC) was low (<6%) for both 20mg doses. Mean PK parameters appeared to be similar if corrected or uncorrected for the pre-dose baseline cortisol concentration and when the PKAP definition for evaluable PK profiles was used.

**Table 2:** Plasma PK variables for DR-HC in healthy subjects in the PK population. All pharmacokinetic variables are uncorrected and presented as mean (s.d.), with the exception of T_max_, which is presented as median (range).

**Parameter**	**5mg^a^**	**15mg^a^**	**20mg^a^**	**20mg^b^**
C_max_ (ng/mL)	82.0 (18.2)	148.8 (29.3)	177.1 (25.5)	178.0 (28.1)
AUC_0−∞_ (h×ng/mL)	562.8 (141.0)	991.6 (162.0)	1180.8 (213.8)	1162.1 (175.7)
AUC_0−12h_ (h×ng/mL)	371.8 (75.8)	770.4 (209.0)	947.7 (174.2)	919.5 (169.7)
T_max_ (h)	0.5 (0.3−1.0)	0.5 (0.3−24.0)	0.5 (0.3−5.0)	0.5 (0.3−5.0)
Terminal half-life (h)	13.7 (8.0)	8.3 (5.1)	6.0 (2.9)	6.7 (3.6)
% extrapolated AUC (%)^c^	17.4 (10.8)	7.5 (6.7)	4.3 (2.6)	5.7 (4.9)

a Test site; ^b^reference site; ^c^percentage of the AUC resulting from extrapolation after the last measurable concentration. AUC, area under the concentration−time curve; AUC_0−∞_, total area under the concentration−time curve; AUC_0−12h_, area under the concentration−time curve to the last sample taken at 12h after dosing; C_max_, maximal serum concentration; PK, pharmacokinetic.

Bioequivalence was demonstrated for the DR-HC 20mg tablets from the test and reference sites for corrected and uncorrected PK parameters, and for both definitions used for evaluable PK profiles (data not shown). Within-subject variability, determined for the two 20mg tablets from different manufacturing sites, was low and below 15% for all examined PK parameters (Table 3).

**Table 3:** Within-subject variability for the two DR-HC 20mg tablets (reference and test sites) in the PK population.

**Parameter**	**Geometric LSMs**		**Within-subject %CV**	**LSM ratio** (test/reference)	**90% CI**
	20mg (reference site)	20mg (test site)			
C_max_ (ng/mL)	175.42	175.56	8.8%	100.1%	96.2−104.1
AUC_0−12h_ (h×ng/mL)	905.05	933.37	10.9%	103.1%	98.3−108.2
AUC_last_ (h×ng/mL)	1088.35	1113.89	11.4%	102.3%	97.3−107.7

AUC, area under the concentration–time curve; AUC_0−12h_, area under the concentration–time curve to the last sample taken at 12h after dosing; AUC_last_, area under the concentration–time curve through to the last measurable concentration; C_max_, maximal serum concentration; CV, coefficient of variation; LSM, least squares mean; PK, pharmacokinetic.

### Dose proportionality

All of the exposure PK parameters for cortisol examined (uncorrected and baseline corrected) were found to be less than dose proportional, with a slope <1, in the 5−20mg dose range (Table 2). The dose-proportionality slopes for baseline-corrected PK parameters (90% CI) were as follows: C_max_, 0.59 (0.52−0.66)h×ng/mL; AUC_0−12h_, 0.74 (0.58−0.89)h×ng/mL; and AUC_last_, 0.79 (0.71−0.87)h×ng/mL. The slope (90% CI) for AUC_0−∞_, which was only calculated for PKAP-defined evaluable concentration−time profiles due to the requirement for an adjusted *R*^2^>80%, was 0.78 (90% CI: 0.70−0.85). Mean plasma AUC_0−∞_ increased 1.8- and 2.1-fold, and C_max_ increased 1.8- and 2.2-fold when the dose of DR-HC increased from 5 to 15mg and 20mg respectively. There were no differences between the two ethnic groups.

### Impact of ethnicity on PK parameters for DR-HC

DR-HC PK parameters were similar for Caucasian and Japanese subjects, with the exception of two outliers, who were both Japanese females and had the lowest body weights recorded among enrolled subjects (48.8 and 52.4kg). Using the two one-sided tests procedure, a significant difference in C_max_ due to ethnicity (group fixed-effect *P*=0.0126 (uncorrected) and *P*=0.0103 (corrected)) was found but, when subject body weight was added to the model, ethnicity lost significance indicating that the between-group differences in PK parameters were explained by differences in body weight.

### Adverse events

DR-HC at all doses was generally well tolerated. AEs were reported in 3/31 patients (10%); all AEs were mild and none were considered by the investigator to be related to study drug. Two subjects reported mild intermittent hiccups considered by the investigator to be possibly related to dexamethasone. Two subjects experienced mild AEs (loose stools, abrasion) that were not considered to be treatment emergent. There were no deaths, serious AEs, or discontinuations due to an AE. There were no clinically relevant changes in laboratory parameters, vital signs, or ECGs.

## Discussion

The aim of glucocorticoid replacement therapy for patients with AI is to mimic endogenous cortisol secretion as closely as possible in healthy subjects in terms of both total exposure and the diurnal cortisol exposure profile (21). Healthy individuals have a very low or undetectable level of cortisol at midnight, which gradually increases to the maximal concentration in the early morning and then declines over the rest of the day (22, 23). Unfortunately, conventional replacement therapy with immediate-release hydrocortisone neither restores nor emulates the intrinsic circadian rhythm of cortisol secretion (1, 24). Current treatment regimens are associated with over- or underexposure of glucocorticoids and the multiple daily dosing required is often associated with poor patient adherence (25, 26).

The novel once-daily, dual-release oral formulation of hydrocortisone investigated in this study was developed to ensure high and robust bioavailability following absorption from the small intestine and proximal colon, thereby providing a predictable cortisol exposure profile over 24h (1). The immediate-release component in the tablet coating provides a rapid rise in cortisol levels after morning oral intake when physiological levels are higher, while the hydrocortisone in the extended-release core is released more slowly so that cortisol concentrations are low when there is low physiological exposure and there is a cortisol-free interval at night, thereby avoiding dose accumulation with repeated doses (1).

Our findings show that once-daily DR-HC provides high exposure during the first 4h, reducing over the remainder of the day and a cortisol-free period during the night. The dual-release tablet therefore mimicked most phases of the physiological cortisol profile, with the exception of the early morning increase that occurs during sleep (22, 23). Our results support those from earlier clinical trials, in which peak plasma cortisol concentrations were observed for the first 4−6h after dosing, with mean T_max_ ranging from 0.63 to 1.11h with the 5 and 20mg doses, followed by a gradual decrease to levels below 50nmol/L (18ng/mL) 18−24h post-dose (15, 16). Furthermore, individual PK parameters for the 5 and 20mg doses evaluated in this study were generally similar to those reported in another PK study in healthy volunteers (15). DR-HC was well tolerated with no safety concerns after single dosing in Caucasian and Japanese healthy volunteers.

Treatment with conventional thrice-daily hydrocortisone is associated with exposure to high cortisol levels in the late afternoon and evening, which may lead to the development of increased cardiovascular risk (10, 12, 27, 28), disturbance of sleep pattern, and cognitive dysfunction (14, 29, 30, 31). By contrast, the afternoon and evening cortisol exposure profile with DR-HC in our study appeared to be similar to the endogenous cortisol profile and, in a previous clinical study in patients with AI, cortisol plasma concentrations following DR-HC declined during the afternoon to significantly reduced levels compared with conventional immediate-release hydrocortisone therapy given thrice daily (16). Hence, the multiple peaks and troughs in cortisol concentration that occur with conventional hydrocortisone therapy are avoided with DR-HC. The beneficial effects of the time−exposure profile of DR-HC have been demonstrated by significantly improved cardiovascular risk factors and metabolic disturbances compared with hydrocortisone three times per day, as well as significant improvements in patient QoL (16).

Studies investigating normal cortisol secretion in healthy children and adolescents (*n*=14) and adults (*n*=33 and *n*=29) have shown that there is a cortisol-free period at night, with cortisol concentrations tending to reach nadir of <50−331nmol/L (<18−119ng/mL) at around midnight (20, 32, 33). Achieving this nighttime cortisol-free period is important from a safety aspect as it allows for repeated dosing without dose accumulation and helps to avoid the detrimental effects of increased nighttime cortisol levels (10, 11, 13, 14). Hence, the target cortisol plasma concentration at midnight in patients receiving glucocorticoid replacement therapy should be similarly low to optimize the clinical outcomes in this population. This is achieved with conventional thrice-daily hydrocortisone therapy (16), and this study indicates that this may also be achievable with once-daily DR-HC. Cortisol plasma concentrations of 0−65.0ng/mL were obtained from the last evaluation of the day at 23:00h, which is in agreement with midnight serum cortisol levels in clinical studies in healthy volunteers (20, 32, 33).

Treatment with DR-HC 20mg seemed to provide higher than endogenous cortisol plasma concentrations during the morning. In the earlier study conducted in AI patients, cortisol exposure during the first 4h post-dose with DR-HC was significantly higher than with conventional thrice-daily hydrocortisone therapy, whereas afternoon, evening, and overall exposure levels were significantly lower (16). Evidence suggests that the detrimental metabolic impact of high plasma cortisol levels in the morning may be minimal, whereas cortisol overexposure in the evening induces a state of insulin resistance and reduced insulin clearance (10). It is thought that the varying effects of morning and evening cortisol overexposure may be related to differences in cortisol clearance and glucocorticoid receptor regulation (10).

Within-subject, day-to-day PK variability in cortisol exposure with DR-HC was less than 15%, probably due to the mechanism of absorption in the gastrointestinal tract. A consistent exposure−time profile within individual patients is a particular advantage of the formulation in the maintenance of glucocorticoid replacement therapy, as it is indicative of its reliability and minimal risk for any absorption failure. As well as minimizing the day-to-day variability in cortisol−time exposure, once-daily dosing is likely to improve patient compliance with therapy compared with multiple-dose regimens (34).

Intersubject variability in both the endogenous cortisol plasma profiles and the cortisol plasma concentrations after hydrocortisone treatment was high in our study, corroborating previous findings (15). The use of a weight-based nomogram for immediate-release hydrocortisone has been shown to reduce intersubject variability in cortisol exposure, as body weight is the main clinical determinant in cortisol clearance rate (35). Hence, the intersubject variability in PK in our study may be at least partly attributed to the wide variation in body weight, mainly resulting from the inclusion of two female Japanese outliers with low body weight, while the majority of remaining subjects were male with markedly higher body weight. Although the nomogram presented by Mah *et al*. (35) cannot be used for DR-HC due to the difference in relative bioavailability versus immediate-release hydrocortisone, it seems rational that application of a similar weight-based nomogram may be useful when administering DR-HC to reduce variability in hydrocortisone exposure between patients, thereby minimizing under- or overtreatment. Ethnicity of the participants did not appear to affect the PK of DR-HC when differences in body weight were controlled for. However, the effects of body weight on the PK of this formulation need to be considered in particular ethnic populations as over- or underexposure to cortisol may occur.

Our study showed that PK exposure parameters for DR-HC were less than dose proportional in the fasted state, which confirms previous findings for DR-HC (15) and is in line with PK data for immediate-release and suspension formulations of hydrocortisone in the same dose range (36, 37). This important PK property of oral hydrocortisone makes it difficult to determine the optimal dosing regimen during intercurrent illness in AI, for which current management is inadequate (1, 7, 9, 38). The recommendation for managing non-severe episodes of intercurrent illness with DR-HC, that is, increasing dosing frequency from once to twice or thrice daily with intervals of 8 ± 2h (i.e. not increasing the morning dose) (39), appears to provide greater cortisol exposure over 24h compared with conventional immediate-release hydrocortisone thrice daily (40). This strategy helps to eliminate the less than dose-proportional exposure seen with increasing doses of hydrocortisone when administered as a single dose.

Plasma cortisol concentrations were measured using LC-MS/MS, a state-of-the-art methodology specific to cortisol (41). Published data on normal serum cortisol profiles are generally based on immunoassays, which lack the selectivity to fully differentiate between cortisol and formed cortisol metabolites. Consequently, plasma cortisol concentrations from immunoassays are higher than those from more selective analytical approaches, such as LC-MS/MS (15). This is an important consideration when comparing the cortisol concentrations reported here with previous data.

In summary, once-daily DR-HC demonstrates a plasma cortisol exposure profile that resembles the normal endogenous cortisol profile for the majority of the day. After a period of high exposure during the first 4h post-dose, there is a gradual reduction in cortisol levels over the afternoon followed by a cortisol-free period at night. As well as confirming previous findings, this study has expanded the PK data to an additional dose and into a different ethnic group, and has demonstrated the low within-subject day-to-day variability of DR-HC, indicating a potential for improved safety versus conventional glucocorticoid replacement therapy. This study has also reaffirmed previous observations that cortisol exposure is less than dose proportional with increasing doses of hydrocortisone, which needs to be considered when managing intercurrent illness in AI. This new formulation of hydrocortisone warrants further investigation in clinical trials in patients with AI.

## Declaration of interest

G J, H L, and S S have financial interests in Plenadren. G J and H L have acted as Consultants for Viropharma/Shire. G J has received lecture fees from Pfizer, Novo Nordisk, and Otsuka, and has also acted as a Consultant for AstraZeneca. C M is an employee at Shire International GmbH, Switzerland, and K R is an employee at Shire PLC, USA.

## Funding

This study was funded by Viropharma (now Shire).
